# Primary Epstein-Barr virus infection presenting with genitourinary symptoms in a sexually active adolescent

**DOI:** 10.1128/asmcr.00241-25

**Published:** 2026-02-24

**Authors:** Sally Lin, Alexandria Martindale, Kelly Waters, Candy Rutherford, Marek Smieja

**Affiliations:** 1 Michael G. DeGroote School of Medicine, McMaster University, Hamilton, Ontario, Canada; 2 Research Institute at St. Joe’s Hamilton 539740 , Hamilton, Ontario, Canada; 3 Department of Laboratory Medicine, Hamilton Health Sciences 3708, Hamilton, Ontario, Canada; 4 Department of Pathology & Molecular Medicine, McMaster University 3710, Hamilton, Ontario, Canada; Rush University Medical Center, Chicago, Illinois, USA

**Keywords:** case report, Epstein-Barr virus (EBV), genital infection

## Abstract

**Background:**

Primary infection with Epstein-Barr virus (EBV) manifests with symptoms of sore throat, fevers, fatigue, and cervical lymphadenopathy. We identified no case reports in the literature of primary EBV infection presenting with genitourinary symptoms, although epidemiologic data suggest that it could be sexually transmitted.

**Case Summary:**

A young, sexually active Canadian woman with recent travel to Central America presented for medical attention with pain on urination, acute painful inguinal lymphadenopathy, rectal pain, and elevated liver enzymes. She was assessed by consultants in Gynecology and Infectious Diseases. She had laboratory and diagnostic imaging to investigate for sexually transmitted diseases and acute hepatitis. A gynecological examination and pelvic ultrasound found no evidence of pelvic inflammatory disease. She tested negative for urinary tract infection and for common sexually transmitted infections including gonorrhea, chlamydia, HIV, syphilis, cytomegalovirus, toxoplasmosis, and hepatitis A, B, and C. We documented a positive heterophile antibody test for mononucleosis, significant vaginal viral shedding of Epstein-Barr virus (EBV)-DNA, and EBV seroconversion indicative of primary EBV infection. Symptoms slowly resolved over 4–12 weeks.

**Conclusion:**

This case presentation demonstrates genitourinary symptoms as an initial clinical manifestation of primary EBV infection and suggests that EBV may be sexually acquired.

## INTRODUCTION

Epstein-Barr virus (EBV) is a double-stranded DNA virus from the Herpesviridae family that latently infects about 90% of adults (1). Primary infection typically occurs in childhood or amongst young adults (1). EBV is primarily transmitted through infected oral secretions, initially replicating in oropharyngeal epithelial cells and subsequently in circulating B-lymphocytes. Infections in preadolescents are often asymptomatic, while infections among teens and adults usually manifest as infectious mononucleosis. In infectious mononucleosis, patients present with sore throat, cervical lymphadenopathy, fever, fatigue, myalgia, an atypical lymphocytosis on blood smear, and subclinical hepatitis with elevated liver transaminases (1). Once infected, viral shedding from epithelial cells can occur for years, and lymphocytes can be latently infected for life (2). EBV also has oncogenic properties; latent EBV infection is linked to lymphoma, nasopharyngeal carcinoma, gynecological malignancy, and lymphoepithelioma-like carcinoma (1–4). However, whether primary EBV infection can infect the female genitourinary tract is not documented in the literature.

## CASE PRESENTATION

**Fig 1:**
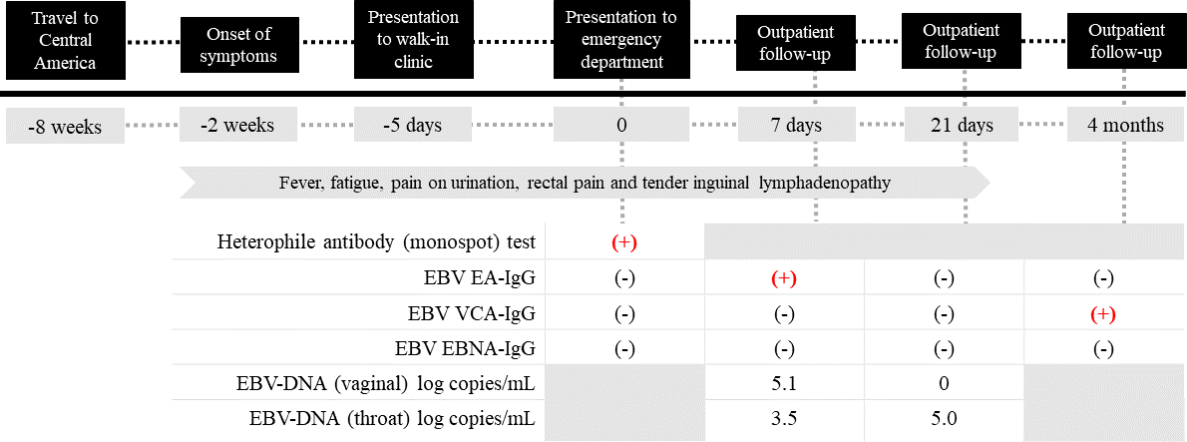
Timeline of the patient’s clinical presentation, including key laboratory results. Because the incubation period of primary EBV infection is usually around 5–6 weeks, EBV likely was transmitted to the patient from her partner in the sexual encounter in Central America (−8 weeks timepoint). The patient developed symptoms at –2 weeks.

A 19-year-old sexually active woman presented to the emergency department (ED) in Hamilton, Ontario, Canada, with a 2-week history of fever, fatigue, pain on urination, rectal pain that was exacerbated by bowel movements, and tender inguinal lymphadenopathy. She was previously well aside from a history of head trauma. She had traveled to El Salvador 6 weeks prior to the onset of symptoms, where she had one male sexual contact that included oral-genital contact (cunnilingus) and unprotected vaginal intercourse. She went to a walk-in clinic 5 days prior and was started on ciprofloxacin for a presumed diagnosis of urinary tract infection, with no relief. She also visited the ED 1 day prior and received 2 g of azithromycin and was started on 500 mg of ciprofloxacin twice daily for suspected pelvic inflammatory disease (PID). A timeline of her presentation and relevant laboratory results is included in Fig. 1.

On physical examination, she was afebrile with stable vital signs. She had no cervical lymphadenopathy or enlarged tonsils and unremarkable cardiovascular and respiratory exams. She had no evidence of hepatosplenomegaly or peripheral rashes. Her pelvic exam revealed two tender lymph nodes in the right inguinal area (1–2 cm), and three palpable tender lymph nodes in the left inguinal area (1 cm). Her initial blood work revealed a leukocyte count of 8.2 × 10^9^/L (reference 4.0–11.0 × 10^9^ L), neutrophils of 1.3 × 10^9^/L (reference 2.0–7.5 × 10^9^/L), and lymphocytes of 5.8 × 10^9^/L (reference 1.5–4.0 × 10^9^/L). Her creatinine was 69 µmol/L (reference 50–98 µmol/L). Her liver enzymes were elevated: aspartate aminotransferase (AST) was 160 U/L (reference: <35 U/L) and alanine aminotransferase (ALT) was 161 U/L (reference: <28 U/L). Atypical lymphocytes were observed on a blood smear.

With her recent travel to Central America and tender inguinal adenopathy, lymphogranuloma venereum (LGV) was considered, and she was seen by Gynecology and Infectious Diseases consultants. Transvaginal ultrasound provided no evidence of PID. Urine culture was negative. A cervical swab and urine sample both tested negative for *Chlamydia trachomatis* and *Neisseria gonorrhoeae* by nucleic acid amplification testing. Additionally, serology for LGV was sent to the National Microbiology Laboratory. In the interim, she was treated with 200 mg of doxycycline daily for 21 days and 500 mg of ciprofloxacin twice daily for 7 days. Serology for LGV was negative at presentation and on repeat testing 6 weeks later. Her serology for *Chlamydia trachomatis* was initially reported as uninterpretable due to cross-reactivity, with identical results 6 weeks later. Serologic testing for HIV, syphilis, cytomegalovirus, toxoplasmosis, and hepatitis A, B, and C was all negative. Hepatitis B vaccination status was unknown. No testing for *Trichomonas vaginalis*, *Mycoplasma genitalium*, or bacterial vaginosis was performed.

Interestingly, despite a lack of oropharyngeal symptoms or cervical lymphadenopathy, a heterophile antibody (monospot) test for infectious mononucleosis was positive at her second ED visit. At the initial Infectious Diseases consultation, her serology was negative for Epstein-Barr Nuclear Antigen (EBNA)-IgG, Early Antigen (EA)-IgG, and Viral Capsid Antigen (VCA)-IgG antibodies. VCA-IgM was not available for testing. At outpatient follow-up 1 week later, her EA-IgG was reactive, while VCA-IgG and EBNA-IgG antibodies remained negative. At her 1-month follow-up visit, her VCA-IgG was indeterminate and her EA-IgG and EBNA-IgG were negative. At her 4-month follow-up visit, EBNA-IgG and EA-IgG were negative; however, her VCA-IgG was now positive.

One week after her initial presentation, a throat swab and a self-collected vaginal swab were tested for EBV-DNA by qPCR (Artus RealStar, Qiagen, Hamburg, Germany), a quantitative method that is validated for plasma specimens but not for mucosal specimens. Swabs were collected in 1 mL of Universal Transport Medium (Copan Italia, Brescia, Italy) and a 200 µL sample was extracted on the easyMag (bioMerieux, St. Laurent, Canada). The throat swab yielded 3.5 log (10) copies/mL and the vaginal swab yielded 5.1 log (10) copies/mL. She appeared to be shedding EBV from the vaginal tract at higher levels than her oropharynx. Repeat swabs 2 weeks later revealed 5.0 log copies/mL in the throat, but no EBV could be detected in the vaginal tract. EBV viral load was not assessed in a urinary specimen.

At her 4-month outpatient Infectious Diseases follow-up visit, her symptoms had largely resolved, with only mild residual tenderness over the inguinal ligaments without palpable lymph nodes. Her liver enzymes and leukocytes had returned to normal. Her VCA-IgG was positive. Based on the seroconversion of VCA-IgG, her positive monospot test, elevated liver enzymes, the presence of EBV-DNA in the vaginal tract at higher concentrations than in her oropharynx, and with the exclusion of plausible alternate causes, we concluded that her infection was due to a primary EBV infection manifesting in genitourinary symptoms and inguinal lymphadenopathy.

## DISCUSSION

We report a case of a young woman with a primary EBV infection, presenting with genitourinary symptoms and rectal pain mimicking LGV or other sexually transmitted infections.

Several case reports have identified EBV as the etiology for acute genital ulcers (5). EBV can be shed in a cell-free infectious form, from B-lymphocytes that were previously infected with EBV (possibly from an oral infection), or from the oropharyngeal epithelium (5, 6). Thus, these findings raise the question of how EBV spreads to the vaginal tract and pelvic nodes.

Shedding of EBV from the female genital tract has been previously documented. EBV can be found in the uterine cervix of approximately 20% of women (6–8), often presenting asymptomatically (9). Individuals with infectious mononucleosis can share the same viral isolates as their sexual partners, and the extent of isolate sharing is much higher in sexual partners than in non-sexual contacts (10). As EBV can be found in the genital epithelium and secretions of both males and females, it may be possible for EBV to be sexually transmitted (9, 10).

To explore the role of sexual transmission in EBV, Crawford et al. conducted a cohort study to determine the risk factors for infectious mononucleosis and seroconversion (11). The study identified a significantly higher seroconversion rate in those who engaged in penetrative sexual intercourse compared with those who engaged in non-penetrative sexual activities including deep kissing (11). Interestingly, condoms played a protective role (though not statistically significant), suggesting that EBV may possibly be transmitted through genital secretions (11). In support of sexual transmission, van Baarle et al. (12) found that EBV type 2 incidence was associated with a higher number of sexual partners. Further, Woodman et al. have linked the acquisition of an incident asymptomatic infection to sexual behavior, with the risk of seroconversion increasing with an increase in number of partners (13). Taken together, these studies suggest that EBV may be transmitted through sexual contact.

Conversely, EBV can also be detected in the uterine cervix of adolescent girls who have never experienced penetrative intercourse (14). Andersson-Ellström et al. showed that sexually experienced teenagers did not have significantly higher levels of EBV-VCA when compared to individuals who had never had sexual intercourse (14). These conflicting studies could be explained by the low amounts of EBV in genital secretions and lack of standardization in their measurement. Viral concentrations in genital secretions were lower than 10 EBV copies/µg of DNA, compared to log (10) 6 copies of EBV/µg of DNA in oral secretions (10). It is also difficult to distinguish transmission through penetrative sexual intercourse from other intimate activities such as deep kissing and cunnilingus (9, 10). Therefore, it is possible that these EBV infections were from either oral or genital secretions (10, 11).

Our report has some limitations, as this is only a single patient. The patient’s negative urine culture specimen was collected after a course of oral antibiotics. However, the patient’s symptoms persisted despite antibiotics, and the rectal pain experienced by the patient would be unusual in a urinary tract infection. We did not test her VCA-IgM serology, which could have added to our diagnosis. Despite the unvalidated use of EBV-PCR from mucosal specimens, we used an assay routinely used for measuring EBV in plasma and found evidence of EBV shedding in the vagina and oropharynx 1 week following her ED visit. This occurred prior to seroconversion, and the potential utility of PCR in early detection of EBV requires further investigation.
